# Stem Cell Niche: iPSC-Based Assembloids for Modeling Human Hematopoiesis

**DOI:** 10.1007/7651_2025_629

**Published:** 2025-01-01

**Authors:** Madeline J. Caduc, Marcelo A. S. de Toledo, Steffen Koschmieder, Simón Méndez-Ferrer

**Affiliations:** 1 Department of Medical Physiology and Biophysics, University of Seville, 41009 Seville, Spain; 2 Instituto de Biomedicina de Sevilla-IBiS ( Hospital Universitario Virgen del Rocío/ CSIC/ Universidad de Sevilla), 41013 Seville, Spain; 3 Department of Hematology, Oncology, Hemostaseology and Stem Cell Transplantation, Faculty of Medicine, RWTH Aachen University, Aachen, Germany; 4 Center for Integrated Oncology Aachen Bonn Cologne Düsseldorf (CIO ABCD), Aachen, Germany; 5 Cambridge Stem Cell Institute, University of Cambridge, Cambridge CB2 0AW, United Kingdom; 6 Department of Haematology, University of Cambridge, Cambridge CB2 0AW, United Kingdom; 7 NHS Blood and Transplant, Cambridge CB2 0AW, United Kingdom

**Keywords:** bone marrow niche, hematopoiesis, microenvironment, induced pluripotent stem cells, assembloids

## Abstract

The bone marrow (BM) niche is a highly specialized and dynamic microenvironment that tightly regulates hematopoiesis in both health and disease. Here, we present a protocol for generating patient-specific 3D BM-mimicking assembloids, which offer precise control over cellular composition and genetic background. This *in vitro* platform enables the dissection of mechanisms underlying hematopoietic regulation and BM niche remodeling. We detail the stepwise differentiation of induced pluripotent stem cells (iPSCs) into hematopoietic and endothelial lineages, the isolation of human primary mesenchymal stromal cells (pMSCs) from femoral heads, and the assembly of BM-mimicking 3D assembloids. Single-cell RNA sequencing of these assembloids identified key myeloid populations and non-hematopoietic lineages such as endothelial cells (ECs) and various MSC clusters, all crucial for stem cell fate determination and niche maintenance. Furthermore, assembloids harboring the JAK2^*V617F*^ driver mutation successfully recapitulated key features of myeloproliferative neoplasms, demonstrating the platform’s potential for mechanistic studies in human hematopoiesis. This approach provides a powerful tool to model both physiological and neoplastic BM niches, facilitating preclinical research and drug development while potentially reducing reliance on animal models.

## 1 Introduction

The 3Rs principles (Replacement, Reduction, and Refinement) have driven the development of innovative research platforms over the past decades, aiming to establish a more ethical framework by minimizing reliance on animal models[1]. While murine models remain the gold standard in preclinical research, they present significant limitations. Notably, mouse models often fail to fully recapitulate the complexity of human diseases[2]. Furthermore, in the era of immunotherapies, the phylogenetic distance between murine and human systems raises concerns about translational relevance, particularly due to variations in antigen processing, immune cross-reactivity, and tumor microenvironment interactions[3]. Therefore, optimizing the use of existing models, incorporating patient-derived data, and developing cutting-edge, human-relevant systems that better reflect cancer biology and immune responses are crucial for improving translational research. The advent of induced pluripotent stem cells (iPSCs) technology has facilitated the generation of patient-specific 2D *in vitro* assays, allowing researchers to study disease mechanisms and therapeutic responses in a controlled setting[4]. However, 2D culture systems fail to replicate the 3D homotypic and heterotypic cellular interactions that occur within complex tissues such as the bone marrow (BM) niche, where spatial organization of key cellular components, including mesenchymal stromal cells (MSCs) and endothelial cells (ECs), is essential for the regulation of hematopoiesis[5, 6]. As a result, the effects of stressors, such as inflammation or drug treatments, on BM cells *in vivo* may differ significantly from observations made in conventional *in vitro* settings, where cells are studied in isolation from their native microenvironment. To bridge this gap, the development of 3D models that recapitulate the organization and complexity of the BM niche is critical for translationally relevant preclinical research. Several approaches have been established to generate BM niche organoids[7, 8], each exhibiting distinct advantages and limitations. The protocol described in this chapter provides a detailed and reproducible methodology for generating patient-specific 3D BM-mimicking assembloids using iPSC-derived hematopoietic and endothelial cells combined with primary MSCs. This fibrin-based model allows precise control over cellular composition and genetic background, facilitating the study of human hematopoiesis and BM niche remodeling. By capturing the heterogeneity of the human BM niche and demonstrating transcriptional homology to healthy and diseased BM, this system serves as a powerful tool for mechanistic studies and preclinical drug testing, while potentially reducing reliance on animal models.

## 2 Materials

### 2.1 Cell Lines (refer to Table 1)

### 2.2 Human Primary MSCs Medium (refer to Table 2)

### 2.3 “Spin-EB” based Protocol for Differentiation of iPSCs toward Hematopoietic Lineages (refer to Table 3)

### 2.4 Differentiation of iPSCs toward Endothelial Cells (refer to Table 4)

### 2.5 Generation of 3D BM-mimicking Assembloids (refer to Table 5)

### 2.6 Chemicals, Peptides and Recombinant Proteins (refer to Table 6)

### 2.7 FACS Antibodies (refer to Table 7)

### 2.8 Other (refer to Table 8)

### 2.9 Software and Algorithms (refer to Table 9)

## 3 Methods

Before starting with the experiments, make sure all required institutional permissions and approvals to work with patient-derived samples were obtained. All biological samples used for the development of the presented protocol were obtained with written informed consent, as approved by the Ethics Committee of the RWTH Aachen University (EK206/09, EK127/12, EK300/13).

### 3.1 Isolation of Primary MSCs

**Figure 1:**
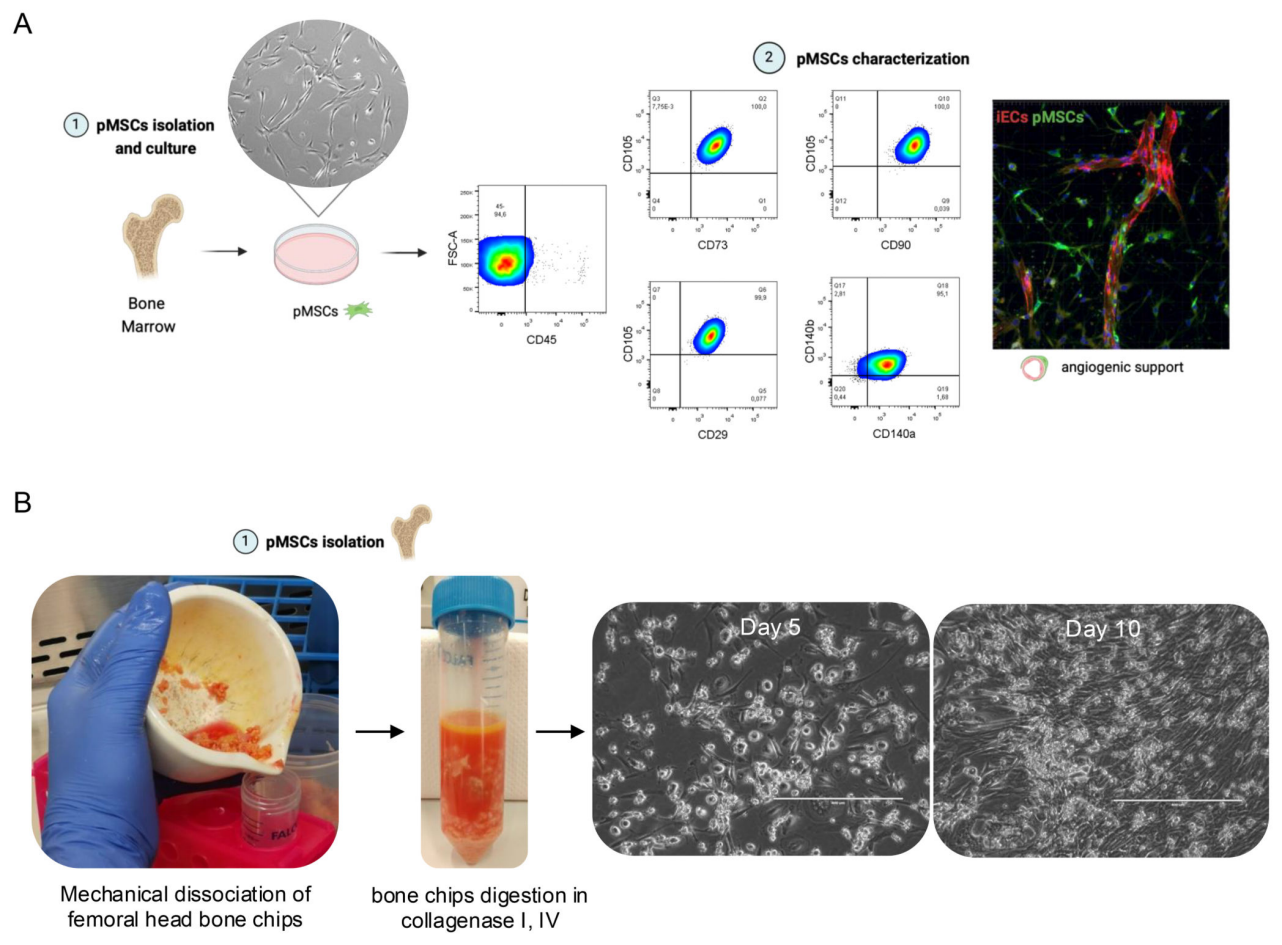
**(A) (1)** Following isolation, pMSCs cultured *in vitro* exhibited a characteristic spindle-shaped, elongated morphology. **(2)** FACS confirmed pMSC identity based on surface marker expression (CD105, CD29, CD73, CD90, CD104a (PDGFRα), CD140b (PDGFRβ)). In a 3D fibrin hydrogel coculture with iPSC-derived endothelial cells (iECs, labeled in red), pMSCs exhibited pericyte-like behavior, aligning along vessel-like structures and supporting vasculogenesis. **(B)** Representative images of the pMSCs isolation process and cell morphology at days 5 and 10 post-isolation. Scale bars: 400 µm.

Primary human mesenchymal stromal cells (pMSCs) were isolated from femoral heads of patients undergoing hip replacement surgery (Department of Orthopedics, Trauma and Reconstructive Surgery, Faculty of Medicine, RWTH Aachen University). The protocol followed was adapted from Stalmann et al.[9] and all procedures were conducted under sterile conditions within a biosafety level 2 (S2) laboratory using a laminar flow hood (Fig.1A).

#### Isolation Procedure

1. Disinfect the laminar flow hood and place a sterile sheet on the working surface.
2. Prepare 1 x 50 mL Falcon tube and 1 x 10 cm cell culture dish containing 10 ml of DMEM (low glucose) supplemented with 10% FCS and 1% Penicillin/Streptomycin (p/s) (referred as pMSC medium).
3. After sterilizing hands and donning sterile gloves, retrieve the femoral head from the sterile recipient using sterile forceps.
4. Harvest spongiosa chips using a sterile curette. Transfer a portion to the 10 cm culture dish containing pMSC medium and the remaining chips to the 50 mL Falcon tube.
5. Discard the femoral head.
6. Mechanically dissociate the bone chips in the culture dish, transferring larger fragments to the Falcon tube for enzymatic digestion (Fig. 1B).
7. Incubate the culture dish in a humidified incubator at 37°C and 5% CO_2_.
8. Add 5–10 ml of Collagenase I, IV mix (1 mg/ml each) to the Falcon tube, depending on the bone chip volume, and incubate at 37°C in a water bath for 45 minutes, shaking vigorously every 15 minutes.
9. Quench enzymatic digestion by adding 10 ml of pMSC medium, then transfer the supernatant (without bone chips) to a fresh 50 ml Falcon tube.
10. Centrifuge at 300 x g for 10 minutes.
11. Resuspend the cell pellet in 5 mL of pMSC medium and seed 2 x 2.5 ml onto 2 x 10 cm culture dishes pre-coated with 7.5 ml pMSC medium (*see* Note 1).

#### Cell Maintenance

12 pMSCs are isolated by adherence selection and expanded until ~80% confluency for passaging.
13 Medium should be replaced every 48 hours, monitoring cell morphology (*see* Note 2) (Fig. 1A-B).
14 Cells are maintained in culture until further use for 3D assembloid generation or cryopreserved in FCS + 10% DMSO until the other cell types are available (*see* Note 3).
15 Depending on the experimental objective, pMSCs can be differentiated into adipogenic, chondrogenic, or osteogenic lineages using lineage-specific differentiation media[9].

#### pMSCs Characterization

The pMSCs used in this study expressed key BM-derived MSCs (BM-MSCs) surface markers, including CD105, CD29, CD73, CD90, PDGFRα, PDGFRβ and provided angiogenic support when co-cultured with iPSC-derived ECs (Fig. 1A)[10, 11]. Flow cytometry was performed on a FACS Canto II (BD Biosciences, San Jose, CA), and data were analyzed using FlowJo software.

1. Wash Cells: Aspirate culture medium and wash adherent MSCs 1x PBS.
2. Add 0.05% Trypsin (enough to cover the cell layer) and incubate at 37°C for 4-5 minutes (monitor for cell detachment by microscopy).
3. Add an equal volume of culture medium containing 10% FCS to inactivate trypsin.
4. Transfer the cell suspension into a 15 ml Falcon and centrifuge at 400 × g for 4 minutes.
5. Remove the supernatant and resuspend the pellet in FACS Buffer to achieve a concentration of ~1 x 10^6^ cells/ml
6. Transfer 100 µL of cell suspension (~1-2 × 10^5^ cells) into a 1.5 mL tube.
7. Add fluorophore-conjugated antibodies at the manufacturer’s recommended dilution. Include appropriate controls.
8. Keep tubes on ice in the dark for 20 minutes.
9. Add 1 ml of FACS Buffer and centrifuge at 400 x g for 4 minutes at 4°C.
10. Discard supernatant and resuspend in 200 µl FACS Buffer
11. Analyze samples in a flow cytometer.

### 3.2 “Spin Embryoid Body (EB)” Differentiation of iPSCs into Hematopoietic Cells

The iPSC clones used for the development of this protocol were reprogrammed from peripheral blood mononuclear cells (PBMCs) of patients with MPN using the CytoTune iPS 2.0 Sendai Reprogramming Kit, following the manufacturer’s instructions. After reprogramming, single-cell iPSCs were seeded, and individual colonies were isolated and genotyped by PCR or allele-specific PCR. PBMCs from MPN patients were obtained from the centralized Biomaterial Bank, Faculty of Medicine, RWTH Aachen University Hospital.

**Figure 2:**
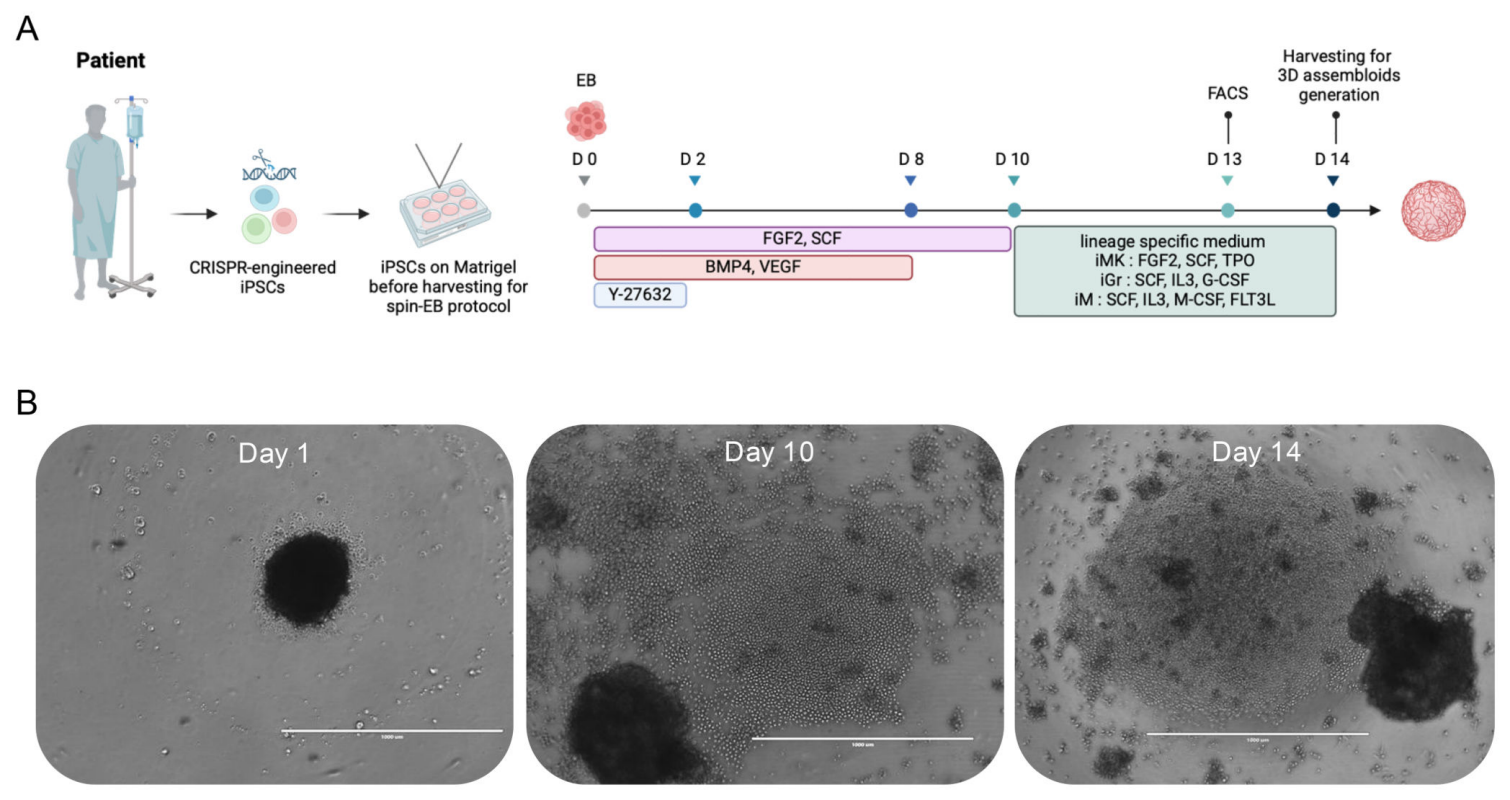
**(A)** Schematic representation of the iPSC differentiation workflow into hematopoietic lineages using the spin embryoid body (EB) protocol. **(B)** Representative bright-field images showing EB morphology at different stages of the differentiation protocol. Hematopoietic cell production typically emerges around day 10, with day 14 showing EB prior to harvesting. Scale bars: 1000 µm.

This differentiation protocol has been previously described [12, 13] and was adapted from Liu et al. [14], utilizing the “spin embryoid body (EB)” method to derive hematopoietic cells, including megakaryocytes, granulocytes, and monocytes/macrophages, from iPSCs. The differentiation process spans over approximately 14 days (*see* Notes 4–6) (Fig. 2A).

#### Cell Seeding

1. Culture iPSCs in 6-well plates coated with Matrigel until they reach 80–90% confluency (Fig. 2A).
2. Pre-warm Accutase to 37°C.
3. Gently aspirate the culture medium from the well.
4. Carefully rinse the well with 1 ml of room-temperature PBS, then aspirate PBS completely.
5. Add 1 ml of pre-warmed Accutase to the well.
6. Incubate at 37°C for 4 minutes, monitoring cell dissociation and rounding under a microscope.
7. Pipette cells gently in the 1 ml of Accutase and transfer to 10 ml of pre-warmed KO-DMEM in a 15 ml Falcon tube.
8. Take 1 ml of KO-DMEM from the Falcon tube, gently rinse the well, and transfer it back to the tube.
9. Centrifuge at 400 x g for 4 minutes and discard the supernatant.
10. Resuspend the cell pellet in 5 ml of KO-DMEM, pipetting gently to avoid bubble formation.
11. Centrifuge again at 400 x g for 4 minutes and remove the supernatant.
12. Resuspend cells in 500 µl of KO-DMEM supplemented with ROCK inhibitor (1:1000).
13. Mix 10 µl of cell suspension with 10 µl of Trypan Blue, and count cells using a hemocytometer.
14. Take the volume of cell suspension corresponding to 500,000 cells and add it to 5 ml of freshly prepared "Day 0" medium.
15. Transfer the suspension into a reservoir compatible with a 96-well multichannel pipette.
16. Using a multichannel pipette, dispense 50 µl of cell suspension in "Day 0" medium per well of a 96-well U-bottom suspension plate.
17. Centrifuge the U-bottom plate at 300 x g for 5 minutes.
18. Examine the plate under a microscope to ensure cells are packed together. If aggregation is insufficient, centrifuge again at 300 x g for 5 minutes (Fig. 2).

#### Daily Medium Change for Spin-EB Differentiation

○ Day 0 – 2: Spin-EB plates are incubated for the first 48 hours without medium change (*see* Note 7) (Fig. 2B).
○ Day 2: Add 50 µl of “Day 2–10” medium to each well.
○ Day 3 onward: Perform a partial medium change, removing 50–85 µl per well and replacing it with 50 µl of fresh “Day 2–10” medium (*see* Notes 8, 9).

Medium Cytokine Supplementation

- Day 2 – 8: Culture in core SFM medium with human albumin (Plasbumin) supplemented with:
  - 10 ng/ml VEGF
  - 10 ng/ml BMP4
  - 10 ng/ml FGF-2
  - 50 ng/ml SCF
- Day 8 onward: BMP4 and VEGF are removed from the medium.

#### Lineage-Specific Differentiation and Pre-Assembly Characterization

Starting on Day 10, daily partial medium changes continue using the core SFM medium with human albumin (Plasbumin), supplemented with:

- Megakaryocytic Differentiation: SCF (100 ng/ml) + TPO (40 ng/ml)
- Granulocytic Differentiation: SCF (100 ng/ml) + IL-3 (30 ng/ml) + G-CSF (100 ng/ml)
- Monocyte/Macrophage Differentiation: SCF (100 ng/ml) + IL-3 (30 ng/ml) + M-CSF (100 ng/ml) + FLT3L (50 ng/ml)

#### iHCs Characterization

On day 13, 8 wells of a 96-well plate per clone per differentiation protocol was harvested for FACS analysis and morphological assessment by cytospins. The resulting iPSC-derived hematopoietic cells (iHCs) included hematopoietic stem and progenitor cells (iHSPC, CD117^+^CD34^+^), iPSC-derived megakaryocytes (iMK, CD45^+^CD61/41^+^), iPSC-derived monocytes/macrophages (iM, CD45^+^CD68^+^), and iPSC-derived granulocytes (iGr, CD45^+^CD66b^+^)[11].

1. Harvest cells from each well using a 1000 µl pipette
2. Filter the suspension through a 100 µm cell strainer to separate iHCs from the EBs.
3. Wash each well with 100–150 µl PBS to collect remaining iHCs.
4. Centrifuge at 350 x g for 5 minutes at 4°C to pellet cells.
5. Carefully remove the supernatant and resuspend cells in ice-cold FACS Buffer.
6. Transfer 1-2 x 10^5^ into 1.5 ml tubes or 5 ml FACS tubes.
7. Add fluorophore-conjugated lineage-specific antibodies at the manufacturer’s recommended dilution. Include appropriate control.
8. Keep cells on ice and incubate for 30 minutes in the dark.
9. Wash cells with 1 ml of ice-cold FACS Buffer to each tube.
10. Centrifuge at 300 x g for 4 minutes at 4°C.
11. Carefully remove the supernatant and resuspend cells in 200-300 µl of FACS Buffer for sample acquisition.

Rest of the cells can be used for morphology characterization by cytospin (*see* Note 10).

1. Pre-wet cytospin membrane with 500 µl PBS and spin at 270 rpm for 5 minutes
2. Load ~1,5-2,5 × 10^5^ cells in 200 µL onto a pre-labeled cytospin slide
3. Spin at 270 rpm for 5 minutes to deposit cells onto the slide e.g. using a Shandon Cytospin 423 cytocentrifuge.
4. Carefully remove membrane without disrupting cell deposition
5. Fix cells with methanol for 4min at room temperature
6. Remove excess by gently tapping on paper towel
7. Air-dry slides before staining.
8. Add 50 µl hematoxylin for 4-5 minutes.
9. Rinse with tap water until excess stain is removed.
10. Dip slides in 1% Hydrochloric acid (HCl) solution (5ml 37% fuming HCl in 200ml distilled water) for a few seconds.
11. Rinse in tap water for 8 minutes, then add 50 µl eosin solution for 1–2 minutes.
12. Wash in distilled water and air-dry slides completely.
13. Mount the slides with Entellan mounting medium and cover with a glass coverslip.
14. Allow slides to dry before imaging.

#### Cell Harvesting for 3D Assembloid Generation

After 14 days of differentiation, the 96-well plates were harvested to isolate iHCs (*see* Note 11) (Fig. 2B). Cell harvesting should be performed immediately before the generation of 3D assembloids. 3D assembloids with a final volume of 100 µl were assembled using the following ratio of iHCs: 30,000 iGr : 30,000 iM : 60,000 iMK.

1. Harvest cells from each well using a 1000 µl pipette, filtering the suspension through a 100 µm cell strainer to separate iHCs from the EBs.
2. Wash each well with 100–150 µl PBS to collect remaining iHCs.
3. Centrifuge at 1000 rpm (200 × g) for 5 minutes in 50 mL Falcon tubes and discard the supernatant.
4. Resuspend the pellet in 1 mL of EBM-2 medium and count cells.
5. Transfer the required number of iMK, iGr, and iM into Eppendorf tubes and store on ice until suspension into fibrin hydrogels together with pMSCs and iPSC-derived endothelial cells (*see* Note 12).

### 3.3 Endothelial Differentiation from iPSCs

**Figure 3:**
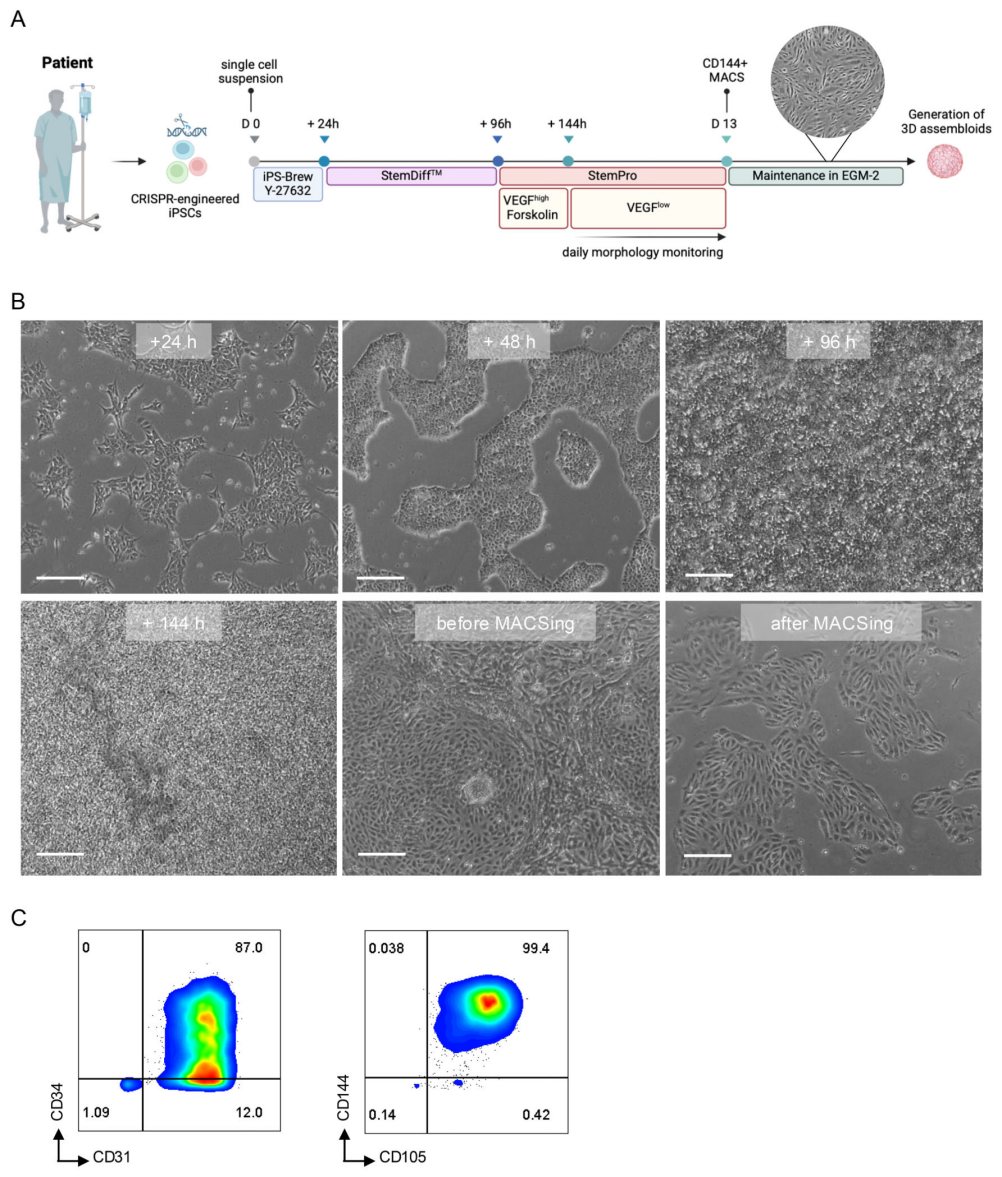
**(A)** Schematic representation of the experimental workflow for iPSC differentiation into endothelial cells (iECs). **(B)** Representative images illustrating culture morphology at different stages of the differentiation protocol. Scale bars: 250 µm. **(C)** FACS box plots illustrating the high purity of the isolated iEC population after CD144+ MACS enrichment, confirmed by key endothelial surface markers CD144, CD31, and CD105.

The protocol for generating iPSC-derived endothelial cells (iECs) was previously described[12] and adapted from Patsch et al.[15]. Differentiation of iPSCs toward ECs occurs over a period of approximately 12 to 14 days (Fig. 3A).

#### Cell Seeding (Day 0)

1. Use feeder-free iPSCs that have undergone at least one passage post-thaw.
2. Before splitting, pre-warm a Matrigel-coated 6-well plate in a 37°C incubator for 30 min, then remove Matrigel.
3. Add 2 ml iPS-Brew with 10 µM ROCK inhibitor (Y-27632) per well.
4. To split iPSCs, remove the medium, rinse once with 1 ml PBS, and add 1 ml of pre-warmed Accutase per well (6-well plate format). Incubate for 4 min at 37°C. Monitor cell dissociation and rounding under a microscope.
5. Transfer the cell suspension to a 15 ml Falcon tube containing pre-warmed KO-DMEM. Rinse the well with 1 ml KO-DMEM and add it to the tube.
6. Centrifuge at 400 x g for 4 min.
7. Remove the supernatant, resuspend the pellet in 500 µl KO-DMEM with 10 µM ROCK inhibitor, and count cells.
8. Seed 3.5 x 10^5^ cells per well in the previously prepared Matrigel-coated 6-well plate containing 2 ml iPS-Brew with 10 µM ROCK inhibitor. Shake the plate gently on the incubator shelf to ensure even cell distribution (*see* Note 13).

#### Mesoderm Induction (96 hours)

9 For the first 96 hours post-seeding, replace the medium daily with 3 ml StemDiff Mesoderm Induction Medium per well (*see* Note 14).

#### Endothelial Induction

10 After 96 hours in StemDiff Mesoderm Induction Medium, switch to endothelial induction by replacing the medium daily with 2 ml StemPro 34 SFM supplemented with 200 ng/ml VEGF and 2 µM Forskolin, along with Supplement, P/S, L-glutamine, and non-essential amino acids per well (*see* Notes 15, 16).
11 After 48 hours in VEGF + Forskolin-supplemented StemPro medium, change the medium daily with 2 ml StemPro supplemented with 50 ng/ml VEGF, Supplement, P/S, L-glutamine, and non-essential amino acids per well. From this point, daily monitoring of EC growth is required to determine the optimal time for MACS enrichment, which should be performed when iECs reach confluency (Fig. 3B).

#### Enrichment for CD144^+^ Cells and iECs Maintenance

12 Following MACS-based enrichment for CD144^+^ cells using CD144 magnetic beads according manufacturer’s protocol, iECs are maintained in 2 ml EGM-2 medium per well in a 0.1% gelatin-coated 6-well plate.
13 Medium is changed daily. When cells reach 90% confluency, they are passaged using Accutase for 4 minutes at 37°C (*see* Note 17,^18^).

#### iECs Characterization

Endothelial differentiation was assessed via FACS using antibodies specific for CD31, CD34, CD105, and CD144 (Fig. 3C).

1. Wash Cells: Aspirate culture medium and wash 1x PBS.
2. Add Accutase (enough to cover the cell layer) and incubate at 37°C for 4 minutes.
3. Add an equal volume of culture medium containing 10% FCS and rapidly transfer the cell suspension into a 15 ml Falcon and centrifuge at 400 x g for 4 minutes.
4. Remove the supernatant and resuspend the pellet in FACS Buffer to achieve a concentration of ~1 x 10^6^ cells/mL
5. Transfer 100 µL of cell suspension (~1-2 x 10^5^ cells) into a 1.5 ml tube.
6. Add fluorophore-conjugated antibodies at the manufacturer’s recommended dilution. Include appropriate controls.
7. Keep tubes on ice in the dark for 20 minutes.
8. Add 1 ml of FACS Buffer and centrifuge at 400 x g for 4 minutes at 4°C.
9. Discard supernatant and resuspend in 200 µl FACS Buffer
10. Analyze samples using a flow cytometer.

### 3.4 Generation of 3D BM-mimicking Assembloids

**Figure 4:**
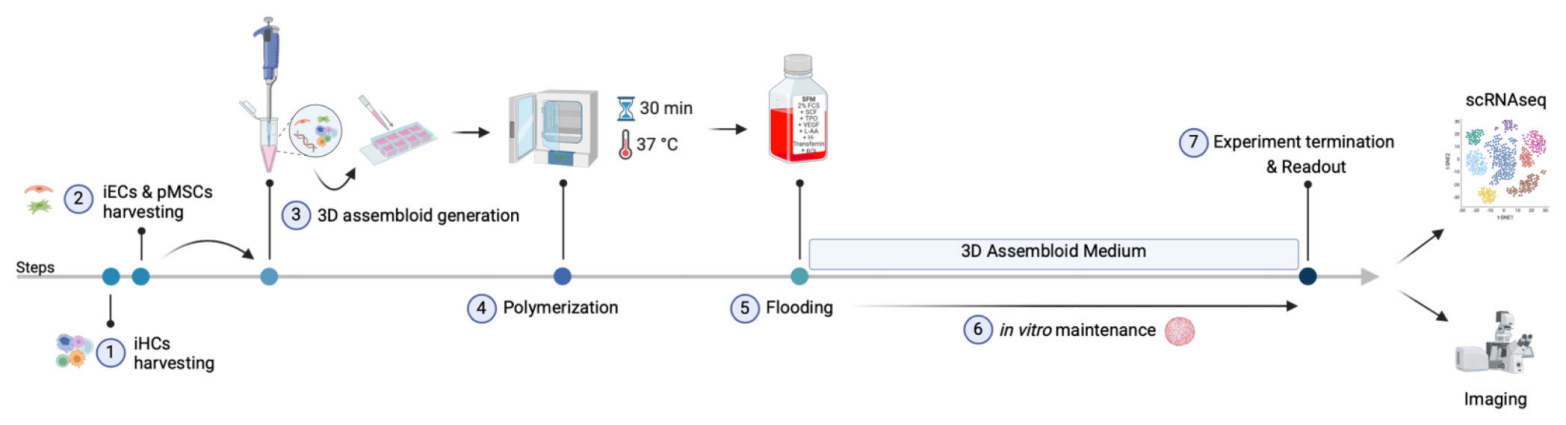
Schematic representation of the protocol for generating scalable 3D BM-mimicking assembloids.

This section details the protocol for generating 3D assembloids by incorporating pMSCs, iHCs, and iECs, obtained as described in Sections 4.1–4.3. Our protocol enables the creation of a 3D BM-mimicking model to study human vasculogenesis and hematopoiesis (Fig. 4)[11].

#### Preparation of Fibrin Hydrogels Solution

Fibrin hydrogels were generated using a modified protocol based on Strassburg et al.[16]. Details on fibrin hydrogel composition are provided in the Materials section.

1. Prior to harvesting the different cell types, prepare working solutions of fibrinogen (5 mg/ml), thrombin (2.5 U/ml), and aprotinin (500 µg/ml) (*see* Note 19). Dilute all components to their final concentrations using a serum-free medium such as EBM-2.
2. Calculate the required volume of each component based on the total number of fibrin clots to be generated.

For a final fibrin clot volume of 100 µl, use the following component ratios:

- Fibrinogen: 50 µl per clot
- Thrombin: 20 µl per clot
- Aprotinin: 5 µl per clot
- Cell-containing fraction: 25 µl per clot

Depending on the platform used for fibrin hydrogel seeding, additional “empty” fibrin hydrogels may be required to secure inserts (e.g., cell crowns) within a 24-well plate or other culture systems (*see* Note 20).

#### Cell Harvesting & 3D Assembloid Generation

3 Harvest iHCs as described in Section 4.2 and store cells on ice until clot assembly (minimize storage time on ice to preserve cell viability).
4 Simultaneously, harvest iECs using Accutase and pMSCs using Trypsin (4 min, 37°C). Monitor cell detachment and rounding using microscopy.
5 Add serum-containing medium (e.g., DMEM low-glucose + P/S + 10% FCS (MSC Medium)). Centrifuge at 400 x g for 4 min.
6 Resuspend cells in serum-free medium (e.g., EBM-2) and count cells (*see* Note 21).
7 Prepare Epitubes for clot formation (one per fibrin hydrogel) and add the required number of iECs and pMSCs:
  1. 100 µl fibrin hydrogel composition:
    1. iECs: 20,000 cells per clot
    2. pMSCs: 60,000 cells per clot
8 Add iHCs (120,000 cells: 30,000 iM + 30,000 iGr + 60,000 iMK) to each Epitube.
9 Centrifuge at 400 x g for 4 min.
10 Remove the supernatant and gently resuspend the pellet in 25 µl serum-free medium (“cell-containing fraction”).
11 Add thrombin and aprotinin, then gently mix (*see* Note 22).
12 Add 50 µl fibrinogen to the Epitube containing the cell suspension, thrombin, and aprotinin, mixing gently 2–3 times.
13 Rapidly transfer the suspension onto the designated experimental platform, ensuring even cell distribution by mixing 2 x up and down.
14 Repeat the process until all fibrin clots are assembled, then carefully transfer the platform to a 37°C incubator for 20–30 minutes to allow polymerization.
15 After polymerization, flood the constructs with the appropriate culture medium and monitor Z-stack formation via microscopy. Return the constructs to the incubator for 3D *in vitro* culture. For details on the 3D Assembloid Medium, refer to the Materials Section.

#### In Vitro Maintenance & Experiment Termination

16 Medium is refreshed every 48 hours, and assembloids are maintained *in vitro* for 5 days while monitoring for retraction. If no signs of retraction are observed, constructs can be cultured longer depending on the experimental setup.
17 Subsequently, assembloids are either:
  - Fixed with 4% PFA and processed for immunofluorescence, or
  - Enzymatically digested for single-cell RNA sequencing (scRNA-seq).

#### Fibrin Hydrogel Digestion for scRNAseq

Fibrin hydrogels were enzymatically digested using a nattokinase-based solution, as previously described[17]. For scRNAseq, single-cell suspensions were processed using the 10x Genomics Chromium system, following the manufacturer’s instructions: ‘Chromium Next GEM Single Cell 3’ Reagent Kits v3.1 User Guide Rev D’.

18 Dissolve nattokinase in 1 mM EDTA (in PBS) to achieve a final concentration of 100 fibrin-degrading units (FU)/ml.
19 Incubate up to 3 fibrin hydrogels in 0.2 ml nattokinase solution at 37°C for 30-45 minutes or until complete gel dissolution is observed. Gently shake the hydrogel-containing aliquot every 10 minutes to ensure uniform enzyme distribution and efficient degradation.

## Figures and Tables

**Table 1:** 

Cell Lines	
Human iPSCs	the iPSC used in the present study were generated in-house, in the Research group of Prof. Dr. Martin Zenke and Prof. Dr. Koschmieder (Department of Hematology, Oncology, Hemostaseology and Stem Cell Transplantation, Faculty of Medicine, RWTH Aachen University, Aachen, Germany)
Primary MSCs	the primary MSCs used in this study were isolated from the femoral heads of patients undergoing hip replacement surgery (Department of Orthopedics, Trauma and Reconstructive Surgery, Faculty of Medicine, RWTH Aachen University)

**Table 2:** 

Primary MSCs Medium	
Name	Final Concentration
DMEM low glucose	
FCS	10 %
Penicillin/Streptomycin	1 %

**Table 3:** 

“Spin-EB” Differentiation	
Name	Final Concentration
iPS-Brew	
Supplement	
Penicillin/Streptomycin	1%
	
**Serum Free Medium (SFM)**	
Name	Final Concentration
IMDM	50 %
Ham’s F-12 Nutrient Mixture	50 %
BSA (only Day 0 for seeding of cells)	0,5 %
Human Plasbumin (from Day 2 on)	0,5 %
Chemically Defined Lipid Concentrate	1 %
GlutaMAX	2 mM
1-Thioglycerol	400 μM
	
**Cytokines to be added to the SFM BSA medium ** **(Day 0 –2)**	
Name	Final Concentration
SFM	
L-Ascorbic Acid	50 μg/ml
H-Transferrin	6 μg/ml
FGF-2	10 ng/ml
BMP-4	10 ng/ml
Y-27632	10 μM
	
**Cytokines to be added to the SFM Plasbumin medium ** **(Day 2 – 10)**	
Name	Final Concentration
SFM	
L-Ascorbic Acid	50 μg/ml
H-Transferrin	6 μg/ml
FGF-2	10 ng/ml
BMP-4 (until Day 7)	10 ng/ml
SCF	50 ng/ml
VEGF (until Day 7)	10 ng/ml
	
**Cytokines to be added to the SFM Plasbumin medium for ** **megakaryocytic differentiation (Day 11 – 14)**	
Name	Final Concentration
SFM	
L-Ascorbic Acid	50 μg/ml
H-Transferrin	6 μg/ml
FGF-2	10 ng/ml
SCF	100 ng/ml
TPO	40 ng/ml
	
**Cytokines to be added to the SFM Plasbumin medium ** **for granulocytic differentiation (Day 11–14)**	
Name	Final Concentration
SFM	
L-Ascorbic Acid	50 μg/ml
H-Transferrin	6 μg/ml
SCF	100 ng/ml
IL-3	30 ng/ml
G-CSF	100 ng/ml
	
**Cytokines to be added to the SFM Plasbumin medium** ** for monocytic differentiation (Day 11 – 14)**	
Name	Final Concentration
SFM	
L-Ascorbic Acid	50 μg/ml
H-Transferrin	6 μg/ml
SCF	100 ng/ml
IL-3	30 ng/ml
M-CSF	100 ng/ml
FLT3L	50 ng/ml

**Table 4:** 

iPSCs Differentiation toward Endothelial Cells	
Medium	
iPS-Brew + supplement + p/s	
KO-DMEM	
StemPro 34 SFM + supplement	
StemDiff Mesoderm Induction Medium	
EGM-2 Endothelial Cell Growth Medium Bulletkit	
	
Cytokines	
Name	Final Concentration
Y-27632	10 μM
VEGF	200 ng/ml
	50 ng/ml
Forskolin	2 μM
SB431542	8 μM
MEM non-essential amino acids	2 μM
L-glutamine	2 mM
	
CD114 magnetic beads for MACSing	

**Table 5:** 

Generation of 3D BM-mimicking Assembloids		
		
**Fibrin Hydrogels Composition**		
Name	Final Concentration	Ratio
Fibrinogen	5 mg/ml	50%
Thrombin	2,5 U/ml	20%
EBM-2		25%
Aprotinin	500 μg/ml	5%
		
**3D Assembloid Medium**		
Name	Final Concentration	
SFM		
FCS	2 %	
SCF	50 ng/ml	
TPO	10 ng/ml	
VEGF	5 ng/ml	
H-Transferrin	6 μg/ml	
Penicillin/Streptomycin	1 %	
L-Ascorbic Acid	50 μg/ml	

**Table 6:** 

Chemicals, peptides and recombinant proteins
Name
Matrigel
Gelatin 0,1%
Fetal Calf Serum (FCS)
Bovine Serum Albumin (BSA)
Human Albumin (Plasbumin)
Dimethylsulfoxid **(**DMSO)
DPBS
EDTA
Penicillin/Streptomycin (p/s)
Accutase
Trypsin
Collagenase I, IV
Nattokinase
Knockout (KO)-DMEM low glucose
Y-27632
Tryptan Blue
Hematoxylin
Eosin
37% fuming hydrochloric acid (HCl)
Distilled water
Entellan

**Table 7:** 

FACS Buffer	
Name	Final Concentration
DPBS	
EDTA	2 mM
Bovine Serum Albumin (BSA)	2 %
	
**FACS Antibodies**	
CD31-PE - clone WM59	
CD34-APC - clone 581 BD	
CD45-APC-Cy7 - REA747	
CD61-FITC - clone VI-PL2	
CD66b-PE - clone REA306	
CD105-FITC – clone 43A4E1	
CD117 (KIT)-PE-Cy7 - clonel 04D2	
CD144-FITC – clone REA199	
CD29	
CD73	
CD90	
CD105	
CD140a (PDGFRα)	
CD140 (PGDFRβ)	

**Table 8:** 

Other
sterile scissors, forceps and curette
cell culture dish (10 cm)
6-well plate
24-well plate
96-well U-bottom suspension plate
p-slide 8 well^high^ ibitreat
cell crowns (home made)
0,22 μm syringe filter
100 pm cell strainer
Cytospin membrane
Glass coverslips
Adhesion microscope slides

**Table 9:** 

Software and algorithms
GraphPad Prism
FlowJo
ImageJ

## Notes

Note 1 If the cell pellet is small, resuspend in 2 ml of medium and seed onto a single 10 cm dish.

Note 2 Isolated pMSCs should exhibit a spindle-shaped, elongated morphology (Fig. 1). A shift to a stellate morphology may indicate activation and a transition to a myofibroblastic phenotype.

Note 3 For 3D assembloid generation, pMSCs should be used at low passages (≤P4–P5) to avoid myofibroblastic bias.

Note 4 Ensure that all materials and media are prepared in advance to minimize processing time, reducing cell stress and sedimentation.

Note 6 From Day 2 onward,

- switch to SFM with human albumin (Plasbumin).
- The core medium for daily medium changes from Day 2 onward can be stored for up to two weeks, but cytokines must be freshly added each day to the required SFM volume for 96-well plate medium changes.

Note 7 Avoid moving the plates within the first 48 hours to prevent disruption of EB formation. By Day 2, EBs should exhibit a defined border, appear light-dense, and show progressive growth over time.

Note 8 To remove medium, tilt the 96-well plate slightly and aspirate from the wall of the well, ensuring that the EB remains undisturbed. Avoid deep pipetting to prevent accidental aspiration of EBs.

Note 9 The volume of medium to be removed varies due to evaporation in the incubator and pipetting inconsistencies. Observe the remaining medium level in each well:

- If 50 µL removal leaves an excessive amount of residual medium, remove up to 35 µL more (total removal: 85 µL).
- Excess residual medium dilutes daily cytokine supplementation, potentially compromising EB differentiation.

Note 10 Considerations to optimize cytospins,

- Ensure a uniform single-cell suspension to avoid clumping during cytospin.
- Optimize methanol fixation time to prevent excessive cell shrinkage.
- Monitor H&E staining times to achieve optimal contrast between nuclei (hematoxylin) and cytoplasm (eosin).

Note 11 Harvesting on Day 14 generally yields high numbers of iHCs. If a higher proportion of uncommitted hematopoietic stem cells (HSCs) is desired, harvesting can be performed on Day 10–12. However, earlier harvesting may result in lower overall hematopoietic cell numbers. In a successful experiment, on day 14 the expected yield is 2 x 10^6^ cells per 96-well plate. However, this may vary depending on the iPSC line and the cytokine conditions used in the differentiation protocol.

Note 12 Depending on cell counts and the experimental design, isolated iHCs can be further enriched for specific hematopoietic lineages using magnetic-activated cell sorting (MACS) and used for additional characterization and functional analyses.

Note 13 Ensure uniform cell distribution across the well. Cell accumulation at the edges may increase cell death upon forskolin treatment.

Note 14 StemDiff Mesoderm Induction Medium may appear yellowish quickly – this is normal. After 24 hours in StemDiff, substantial cell debris is expected. Robust cell expansion occurs after 96 hours of incubation in StemDiff.

Note 15 Prepare stock solutions of StemPro 34 SFM supplemented with Supplement, P/S, L-glutamine, and non-essential amino acids. Freeze 40 ml aliquots. VEGF and Forskolin should be added freshly immediately before medium change.

Note 16 After 24 hours of VEGF + Forskolin treatment, cells may detach in patches. To avoid cell loss, handle plates gently during transfer from the incubator and medium changes.

Note 17 CD144^+^ cells can be cryopreserved in FCS supplemented with 10% DMSO. Efficient thawing and recovery should be performed in EGM-2 medium on gelatin-coated 6-well plates.

Note 18 For 3D Assembloid generation, EGM-2 medium was supplemented with 8 µM TGF-β inhibitor (SB431542) to prevent cell senescence and only low-passage iECs (≤ passage 4) were used.

Note 19 To ensure rapid polymerization, store the fibrinogen working solution in a 37°C water bath until use for clot assembly.

Note 20 In this study, different platforms were used for fibrin hydrogel seeding, depending on the experimental requirements. For optimal optical quality in confocal microscopy imaging, the µ-slide 8 Well High ibiTreat was used, requiring a fibrin hydrogel volume of 200 µl. When using homemade cell crowns (fabricated from the base of a 100 µl pipette tip), a fibrin volume of 100 µl was used. Volumes of fibrin components and cell numbers should be adjusted accordingly based on the platform and hydrogel size.

Note 21 For live-cell tracking, fluorescent cell trackers such as PKH dyes can be used to label cells and monitor their dynamics during *in vitro* cultivation. Ensure that labeling conditions do not compromise cell viability or functionality, and optimize dye concentrations accordingly.

Note 22 From this step forward, work quickly to prevent cell activation and sedimentation. If using cell inserts pre-glued into a well plate, ensure they are pre-positioned under a laminar flow hood while cells are being centrifuged. For rapid handling, use two pipettes simultaneously. In case of a 100 µl fibrin hydrogel volume: 1 x 50 µl pipette to add fibrinogen to each Epitube + 1 x 100 µl pipette to seed the fibrin hydrogel onto the chosen platform.
